# Psychiatry on fire: Climate change and the role of mental healthcare

**DOI:** 10.1192/j.eurpsy.2024.745

**Published:** 2024-08-27

**Authors:** F. Poukhovski-Sheremetyev

**Affiliations:** Psychiatry, McGill University, Montréal, Canada

## Abstract

**Introduction:**

What is the psychiatrist’s role on a burning planet? As our world faces the existential ramifications of irreversible climate change, clinicians are contending with what purpose a normalizing institution like psychiatry can have in increasingly abnormal times.

**Objectives:**

This presentation investigates the role of the modern mental health clinician by examining psychiatry’s current impotence in the face of climate crisis. It will be shown that current approaches are often complicit in psychiatry’s historical depoliticization of mental health and subsequent individualization of social concerns. It will be argued that the only way psychiatry can maintain its ethical obligations to its patients is by taking a courageous sociopolitical stance.

**Methods:**

Emerging from a multidisciplinary literature review on the relationship between psychiatry and social crises, this work examines our field’s response to climate change in particular. A focus is made on literature that explores psychiatry’s political obligations, current trends in climate psychiatry, and proposed social psychiatric approaches to the climate crisis.

**Results:**

It is shown that while ecological collapse tangibly affects our patients, psychiatry often fails to engage socio-politically with the crisis’ root causes. Framing intense reactions to climate change as trauma responses and developing neo-diagnoses such as “ecoanxiety” both risk individualizing inherently social experiences. However, Psychiatrists are also uniquely positioned to speak with authority about social crises and to articulate what a more comprehensive medical response to climate change might look like.

**Conclusions:**

Given climate change’s disproportionate effects on disenfranchised populations, it is increasingly clear that health is inextricable from social circumstances. As a result, political inaction is incompatible with our ethical duty to serve patients’ health, both in the clinic and beyond it.

**Disclosure of Interest:**

None Declared

